# Phenotyping the Sheep Tail: Histological Depiction of Caudal Spine Structures in Sheep

**DOI:** 10.1111/ahe.70022

**Published:** 2025-02-06

**Authors:** Hannah Hümmelchen, Henrik Wagner, Sabine Wenisch, Sven König, Axel Wehrend

**Affiliations:** ^1^ Veterinary Clinic for Reproductive Medicine and Neonatology Justus‐Liebig University Gießen Germany; ^2^ Institute of Veterinary Anatomy, Histology and Embryology Justus‐Liebig University Gießen Germany; ^3^ Institute of Animal Breeding and Genetics Justus‐Liebig University Gießen Germany

**Keywords:** histology, sheep, tail

## Abstract

The morphology of sheep tails remains relatively understudied. Given the escalating discourse on routine removal of the caudal spine in sheep, a thorough exploration of the anatomical structures within this region is imperative. To examine the tails in detail, this study undertook histological characterisation of three segments (the cranial, middle, and caudal segments) of the tails of 12 undocked Merino sheep lambs. Six lambs were selected for having short tails (37.1 ± 3.0 cm) while the remaining six were chosen for their long tails (49.4 ± 1.7 cm). Immunohistochemical labelling using neuron‐specific enolase antibodies was performed to examine the nerve structures in the tail tip. The general structure of the skin resembles that of other domestic mammals. Interestingly, many sweat and sebaceous glands were found in all three tail segments. These findings support the hypothesis that the tails of sheep play a significant role in thermogenesis and perhaps olfactory communication. The study also revealed nerve fibres extending to the tip of the tail. This observation supports the requirement for pain elimination during tail tip amputation.

## 1 Introduction

Domestic sheep naturally possess short tails that have developed into longer woollen appendages through selective breeding. With the waning economic importance of wool, tail docking has become a global norm in sheep husbandry (Hümmelchen et al. 2022). This practice primarily aims to prevent maggot infestation in the tail region caused by faecal contamination (Wall 2012). In some countries, tail docking of sheep is severely restricted or prohibited (Hannemann et al. 2017). When sheep tails remain intact, variations in tail lengths within a population become evident. At birth, the tail lengths of Merino sheep range from 17.0 to 28.2 cm (Hümmelchen et al. 2023).

The sheep tail has multiple functions, albeit with significantly reduced movement compared with that of other animal species. Observations have indicated that the tail is raised when the animal feels threatened, during flight, defecation, or urination. Additionally, lambs exhibit wagging movements while seeking teats during milk intake, whereas adult sheep use tail movement to repel insects (Kiley‐Worthington 1976). Therefore, amputation or partial removal may limit the expressive abilities and defensive gestures of these animals. To comprehensively assess the advantages and disadvantages of tail amputation, detailed examination of the anatomical structure of the sheep tail is crucial.

A recent study focused on the number of vertebrae and the occurrence of vertebral abnormalities in the tail region of Merino sheep, and the significance of these findings for the avoidance of docking (Hümmelchen et al. 2023b). However, a comprehensive histological‐anatomical account of the structures within the caudal vertebral column in sheep does not exist. Some anatomical textbooks only outline the fundamental structures of tails across different animal species (König and Liebich 2015; Reece and Rowe 2017; Salomon 2015a, 2015b, 2015c). For investigation of the sheep tail in greater detail, this study involved the histological examination of three segments (the cranial, middle, and caudal segments) of the tails of undocked Merino sheep lambs.

## 2 Materials and Methods

### 2.1 Animals

All investigations were approved by the competent Animal Welfare Commission (V 54—19c 20 15 h 01 GI 18/14 No. G 44/2021).

In this study, the tails of 12 undocked Merino lambs were examined. The animals were slaughtered for commercial purposes, and their tails were removed at the sacrum. Documentation of the slaughtered lambs included sex, tail length, and tail circumference, as presented in Table 1. The animals were selected according to their tail lengths: the six animals with the shortest and the six with the longest tails were chosen.

**TABLE 1:** Tail length, circumference, and selection criteria of the examined animals in centimetres (cm).

Animal number	Tail length	Tail circumference	Selection criterion
1	35.0	12.5	Short
2	53.0	16.0	Long
3	41.3	14.0	Short
4	48.5	12.0	Long
5	48.0	12.5	Long
6	49.0	14.0	Long
7	37.0	13.0	Short
8	37.0	12.0	Short
9	32.2	11.5	Short
10	50.0	14.0	Long
11	48.0	14.0	Long
12	40.0	14.0	Short

The lambs were born at Oberer Hardthof, a teaching and research centre affiliated with Justus‐Liebig University Gießen. They were weaned from their mothers at 10 weeks of age and slaughtered after reaching the required weight at 14 weeks of age. The lambs were kept in individual pens alongside their mothers for the first 4 days, after which they were assigned to small groups. Beginning in the second week of life, they were provided hay sourced from Oberer Hardthof and concentrated feed from Raiffeisen (RWZ Schaf 18 Uni Press).

The tail length and circumference were measured in the live animals using a suitable measuring device. Further detailed information on the measurement methods can be found in Hümmelchen et al. (2023).

### 2.2 Tissue Preparation and Histological Examination

Immediately after removal, the tail was shaved on site using a disposable razor. The tails were then divided into three predefined sections. Sections at the level of the base of the tail, the middle section and the tip of the tail were about 0.3–0.5 cm thick and were made by the same person using a diamond pathology saw (Cut‐grinder, 0.2 mm diamond saw blade, patho‐service GmbH, Oststeinbek, Germany). To ensure proper fixation, the tissue specimens were stored in 4% paraformaldehyde (PFA) (Carl Roth, Karlsruhe, Germany) and 0.1 M sodium dihydrogen phosphate (NPP) (Carl Roth, Karlsruhe, Germany) at 8°C for up to five weeks. Then decalcification was conducted at room temperature (20°C–22°C) over a 7–12‐day period, using a solution consisting of TRIS Pufferan (Carl Roth, Karlsruhe, Germany) and ethylenediaminetetraacetic acid disodium salt dihydrate (EDTA) (Sigma‐Aldrich/Merck, Darmstadt, Germany). Subsequently, the sections underwent a four‐day dehydration process using 2‐propanol (Carl Roth, Karlsruhe, Germany) and were embedded in paraffin (Roti‐Plast, Carl Roth, Karlsruhe, Germany). After embedding of the specimens, histological cross sections of 5 μm thickness were prepared by using a rotational microtome (RM 2265, Leica, Germany) and stained using a haematoxylin–eosin solution (haematoxylin solution according to Gill II, Carl Roth, Karlsruhe, Germany; Eosin G solution 0.5%, aqueous, Carl Roth, Karlsruhe, Germany; acetic acid 100%, Merck, Darmstadt, Germany; hydrochloric acid 25%, Carl Roth, Karlsruhe, Germany).

For immunohistochemistry, histological cross‐sections of the tail tip were incubated for 30 min at room temperature (20°C–22°C) with the neuron‐specific enolase (NSE) antibody (NSE Clone 5E2, Monoclonal Mouse Merck/Chemicon MAB324) and dilution buffer (Dako S3022). In the next step, the sections were stored for 20 min at room temperature (20°C–22°C) in a polymer system (Dako EnVision + System‐HRP Anti‐Mouse, Dako K4001) and a further five minutes in a peroxidase substrate (Vector‐Linaris, Vector Nova Red Peroxidase Substrate Kit). The sections were subsequently counterstained with haematoxylin (Shandon Instant Haematoxylin Kit, Fisher Scientific, Schwerte, Germany).

### 2.3 Qualitative Analysis of the Histological Tissue Sections

**FIGURE 1:**
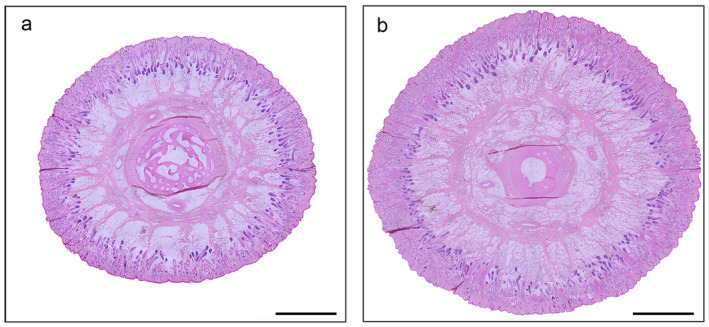
Overview of the histological cross‐section of the caudal tail section of a long‐ (a) and a short‐tailed (b) 14‐week‐old Merino sheep (HE) (50× magnification; bar = 2,6 mm).

Using a light microscope (Photomicroscope Axiophot Zeiss, Oberkochen, Germany) with a connected camera and the software from Leica Microsystems (Ltd., Heer Brugg, Switzerland Leica DFC 320), for each animal all three locations of the tails (cranial, middle, and caudal) were examined histologically and the results were subsequently photo‐documented. However, NSE immunohistochemistry was only carried out on samples from the tip of the tails to check whether the innervation extends to the region of the tips in both subgroups. The overviews of the histological cross‐sections of the caudal tail section of a long‐ and a short‐tailed 14‐week‐old Merino sheep shown in Figure 1 were made with the microscope Leica DM 2500 LED and the connected camera Leica DMC 4500.

## 3 Results

### 3.1 Histological Structure of the Lamb's Tail

The general structure of the skin in the tail area of lambs and its composition closely resembled those of other mammalian skin.

The separate layers of epidermis, dermis, and subcutis were clearly visible.

**FIGURE 2:**
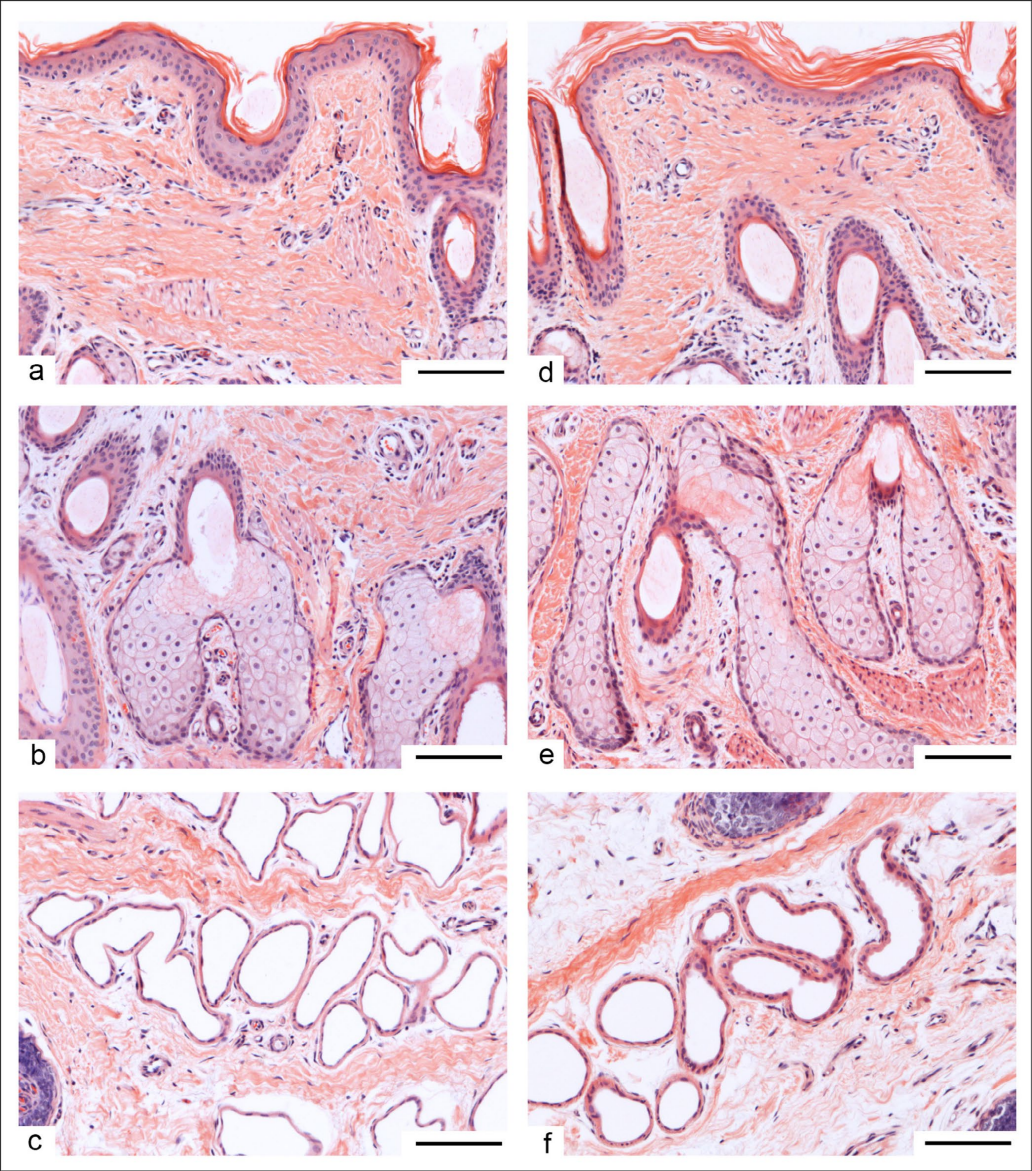
Different sections of the tail of a long‐ (a–c) and a short‐tailed (d–f) 14‐week‐old Merino sheep (HE) (125× magnification; bar = 100 μm). (a, d) Overview of the epidermis. (b, e) Holocrine sebaceous gland adjacent to a hair follicle displaying nuclear pyknosis. (c, f) Tubular sweat gland cross‐sections.

The epidermis displayed a characteristic keratinised, multilayered squamous epithelium, predominantly composed of keratinocytes, commencing with a stratum basale comprising basophilic cuboidal and columnar cells. Above this level, the stratum spinosum, with two to three cell layers, led to a stratum granulosum consisting of one to two cell layers. Finally, a multilayered stratum corneum with distinct exfoliation was observed (Figure 2a,d).

Within the stratum papillare, loose connective tissue containing glands, numerous hair follicles, and sections of blood vessels and nerve fibres were observed.

The exo‐epithelial glands located in the connective tissue beneath the epithelium were identified as holocrine sebaceous glands encircling hair follicles. Figure 2b,e illustrate the transformational process of a sebaceous gland towards secretion, marked by nuclear pyknosis.

The reticular layer contained hair roots embedded within collagenous connective tissue, along with numerous tubular sweat glands (Figure 2c,f). Due to their convoluted shape, multiple sections of the tubular glands were situated next to each other. A single‐layered cuboidal epithelium lined the walls of the sweat glands.

Blood vessels were distinguishable based on their vascular endothelium. They could be categorised into venules and arterioles. In several middle and caudal tail sections, the *arteria* and *vena caudalis mediana* were distinctly visible and identifiable in the ventral region.

Nerve fibres were found in the neighbouring tissues.

Various sections of striated skeletal muscle with peripheral cell nuclei were visible beneath the adipose tissue.

The vertebral bone exhibited lamellar bone characteristics with densely packed collagen fibres. Remodelling during chondral ossification was evident in four of the tails. It was observed in the caudal tail sections of three tails, and in the middle section of one tail. Cartilage was absent from the tail tissues of the remaining animals.

### 3.2 Comparison of the Histological Structures in the Cranial, Middle and Caudal Tail Sections

The epidermis, dermis, and subcutis were consistent across the cranial, middle, and caudal tail sections. Hair follicles containing holocrine sebaceous glands and abundant sweat glands were present in all segments. However, a notable decline in muscle development and vertebral bone thickness was observed towards the tail tip. Bone tissue was absent from the caudal tail sections of three animals.

Nerves were consistently present in all sections; however, their quantity and size varied among individual animals. Significant amounts of fatty tissue were evident only in the cranial tail sections. Figure 2 shows a comparative illustration of the different structures in a long‐ (Figure 2a–c) and a short‐tailed (Figure 2d–f) 14‐week‐old Merino sheep.

### 3.3 Histological Structure of the Lamb's Tail Examined Using NSE

**FIGURE 3:**
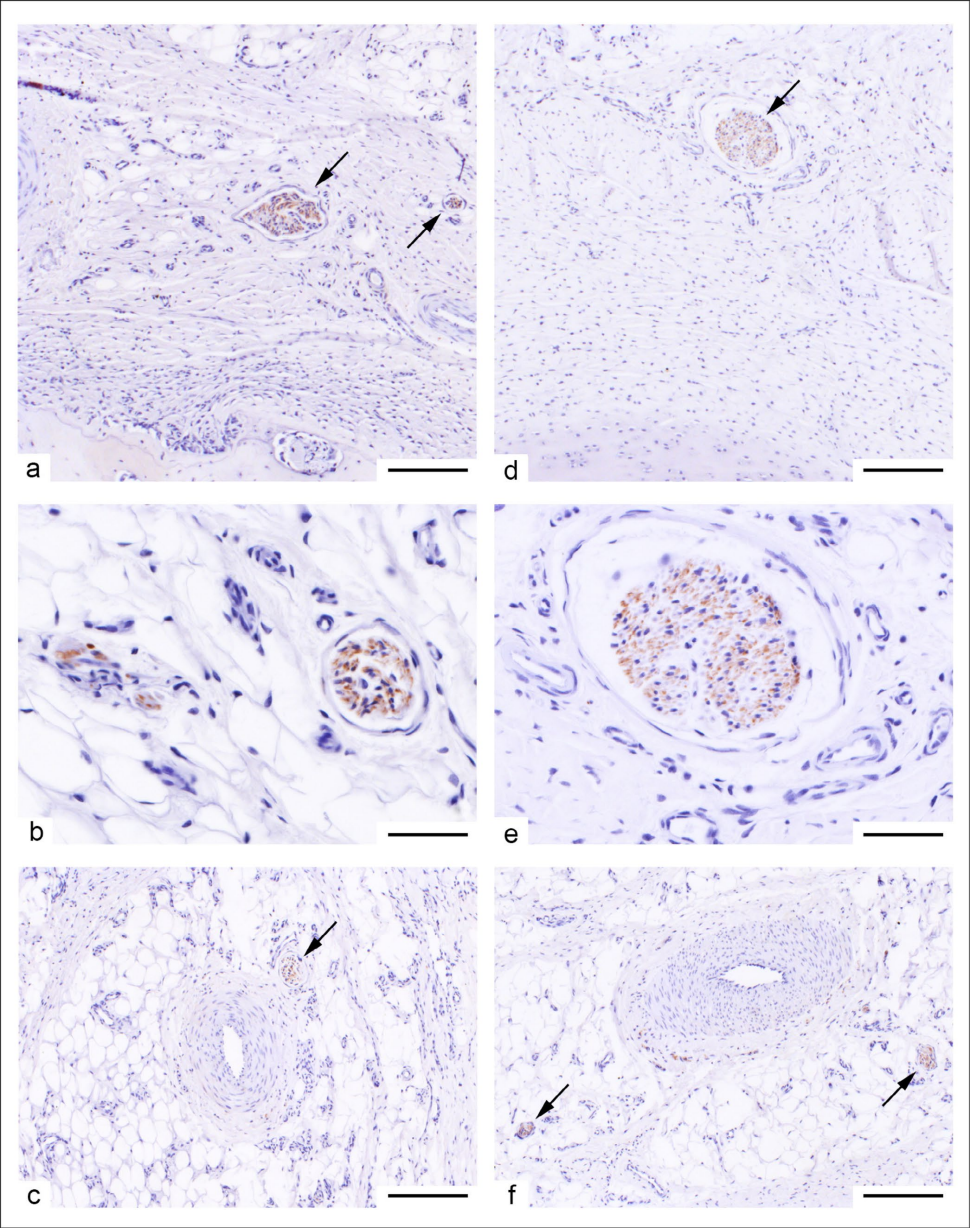
Cross‐sections of tail tips using NSE immunohistochemistry of a long‐ (a–c) and a short‐tailed (d–f) lamb. (a, d) Overview of the subcutis with transversely cut nerves (arrows) of different diameters. Striated muscle and fatty tissue can be seen in the connective tissue while bone tissue (caudal vertebrae) is located at the lower edges of the figures (62,5 x magnification; bar = 275 μm). (b, e) Detailed magnification of nerve cross‐sections located within the subcutis (250× magnification; bar = 50 μm). (c, f) Arteries of the subcutis. Dot‐like patterns of marked vegetative nerve fibres can be seen along the outer arterial wall within the adventitia. Marked nerve fibres (arrows) and fatty tissue are located in the vicinity of the arteries (62,5× magnification; bar = 200 μm).

Nerve fibres visualised using the NSE antibody were clearly visible as shown in Figure 3 in cases of the tip of a long‐ (Figure 3a–c) and a short‐tailed (Figure 3d–f) lamb.

Most of them were cross‐sections of nerves containing myelinated and non‐myelinated fibres. The nerves were located in the connective tissue, in the immediate vicinity of striated muscles (Figure 3a,b,d,e). In addition, punctiform labelling was visible in the adventitia of arteries (Figure 3c,f), which refers to the vegetative innervation of the vessels. In sum, no differences in innervation between the long‐ and short‐tailed lambs could be detected.

## 4 Discussion

Tail amputation in sheep is a global practice (Hümmelchen et al. 2022). Despite its widespread occurrence, comprehensive investigations elucidating the histological architecture of the sheep's tail, which provide information about its physiological role, are lacking. Consequently, the precise distribution and abundance of crucial anatomical structures such as glands and nerve fibres remain unexplored. The functional roles of an organ are often based on its histological composition. The tail of the sheep is conventionally associated with roles encompassing balance, communication, and insect repellence (James 2006). Moreover, if sufficiently elongated, the tail offers a protective cover for the anogenital region (Kiley‐Worthington 1976). However, these assertions predominantly rely on expert opinions, lacking robust empirical support and comprehensive scientific validation (Fisher et al. 2004). The restricted mobility of the sheep tail compared with that of other animal species is conjectured to be influenced, in part, by the substantial wool covering (Kiley‐Worthington 1976).

In this study, blood vessels, sebaceous glands, and sweat glands were evident in all tail sections. However, the role of sweat glands in the tail region remains unclear. These glands, which are akin to other sheep‐specific glands (Ganter 2019), may facilitate intraspecific communication and tail amputation could hinder this communicative aspect. Moreover, sweat gland development is important for skin protection and heat dissipation (de Amorim et al. 2019). The significance of thermoregulation in woollen sheep is crucial, especially during periods of heat stress, when the insulating properties of wool limit the thermal contribution of the body surface. Several studies have explored the effects of tail docking on thermoregulation in fat‐tailed sheep, demonstrating an improvement in post‐docking thermoregulation. This effect has been attributed to enhanced air circulation around the hindquarters (Hafez, Badreldin, and Sharafeldin 1956). Another hypothesis suggests that a thicker layer of subcutaneous fat in docked fat‐tailed sheep enhances thermoregulation efficiency (Juma, Gharib, and Eliya 1971). However, studies in wool sheep breeds without fat tails are currently lacking. According to our hypothesis, tail docking may adversely affect thermoregulation in wool sheep. Future studies should explore the potential role of the tail in sheep thermoregulation.

Histological examination revealed that the nerve supply extended to the tail tip. In a study on different docking lengths, no results were available with regard to the nerve structure of the amputated tail (Götz, Mendel, and Gayer 2023). Unfortunately, there are no further details on the study methodology.

The neuron‐specific enolase (NSE) antibody used in this study is known as a marker for neurons and neuroendocrine cells. In human medicine, it is used to detect neuronal injury and neuroendocrine tumours (Yuan et al. 2024). NSE antibodies are also suitable to detect central and peripheral nervous tissue in meat products (Lücker et al. 2000). Therefore, this antibody was chosen as a first step to examine the innervation pattern of the tips of long‐ and short‐tailed lambs. It can be assumed that the nerve fibres detected in the histological sections represent branches of the spinal nerves composed of visceroefferences and somatoefferences. The filum terminale of the cattle is described to extend only as far as the caudal section of the 4th sacral vertebra (Nickel, Schummer, and Seiferle 1992) and similar conditions probably exist in sheep. However, it cannot be ruled out that its terminal branches extend into the tail tip region of the lambs. Based on the observed innervation patterns, no differences can be identified between the tails of long‐ and short‐tailed lambs.

Therefore, the present findings justify the assumption that tail amputation in 14‐week‐old sheep induces pain irrespective of the amputation site. This questions the assertion that pain is reduced when amputating the tail in the caudal area, which was determined in a study on pain experienced by lambs when the tail is amputated at a length of 15 cm (Götz, Mendel, and Gayer 2023).

In view of the innervation patterns the findings suggest that analgesia is required even if only the tip of the tail is to be amputated.

## 5 Conclusion

Histological examination of the tails of short‐ and long‐tailed lambs revealed nerve fibres, sweat glands, and sebaceous glands up to the tip of the tail. This leads to the conclusion that the tail may have various physiological functions, and that tail amputation is associated with pain, also in the area of the tail tip.

## Conflicts of Interest

The authors declare no conflicts of interest.

## Data Availability

The data present in this study are available upon request from the corresponding author.
